# Magnetic Phase Transition in the Quasi-One-Dimensional Spin Chain System Fe_0.75_Cu_0.25_NbO_4_

**DOI:** 10.1021/acsomega.5c06809

**Published:** 2025-12-12

**Authors:** Diego da Silva Evaristo, Romualdo Santos Silva, Raí Figueredo Jucá, Cledson dos Santos, Gilberto Dantas Saraiva, Javier Gainza, João Elias Rodrigues, Eva Céspedes, José Luiz Martínez, José Antonio Alonso, Igor Frota de Vasconcelos, Francisco Gilvane Sampaio Oliveira, Nilson dos Santos Ferreira, Antônio Joel Ramiro de Castro, Edson Caetano Passamani, Marcelo Andrade Macêdo

**Affiliations:** † Departamento de Física, 74391 Universidade Federal de Sergipe, São Cristóvão, SE 49100-000, Brasil; ‡ Faculdade de Educação, Ciências E Letras Do Sertão Central, Universidade Estadual Do Ceará, Quixadá, CE 63902-098, Brasil; § 16379 Instituto de Ciencia de Materiales de Madrid, CSIC, Madrid, Cantoblanco 28049, Spain; ∥ European Synchrotron Radiation Facility (ESRF), 71 Avenue des Martyrs, Grenoble 38000, France; ⊥ Department of Engineering and Material Sciences, Technology Center, Federal University of Ceará, Campus do Pici, Bloco 729, Fortaleza, CE 60440-900, Brazil; # Universidade Federal do Ceará, Campus Quixadá, Quixadá, Ceará 63902-580, Brasil; ∇ Departamento de Física, 28126 Universidade Federal do Espírito Santo, Vitoria, ES 29075-910, Brasil

## Abstract

In the present work, we conducted a comprehensive study using multiple experimental techniques to elucidate the structural, charge-state, vibrational, and magnetic properties of the quasi-one-dimensional spin chain system Fe_0.75_Cu_0.25_NbO_4_. We found that substituting Fe^3+^ (3d^5^, s = 5/2) with Cu^2+^ (3d^9^, s = 1/2) induces significant changes in the lattice parameters, mixed valence states of Cu (Cu^2+^ and Cu^1+^), and oxygen vacancies, as well as a ferrimagnetic (FiM) ordered state at room temperature. The FiM state is supported by double-exchange interactions between Fe^3+^ and Cu^2+^, mediated by oxygen, which highlight the magnetization mechanism and help describe the complex magnetic interactions among the Fe ions in this compound. We examined the temperature dependence of the magnetic order and, notably, observed a transition from ferrimagnetic to antiferromagnetic (FiM to AFM) at T = 38.4 K. ^57^Fe Mössbauer spectroscopy indicates two Fe species, both in the trivalent state: one arising from Fe ions in a distorted octahedral configuration that interact with octahedrally substituted Cu polyhedra, favoring a FiM state among Fe ions with an ordering temperature above 300 K, and a second species associated with conventional Fe–Fe interactions that lead to the ordinary AFM state at 38 K. The magnetic transition was confirmed by the *M vs T* and *ΔS*_*M*_ *vs T* curves. Furthermore, Raman spectroscopy captures this magnetic transition and reveals clear spin-phonon coupling.

## Graphical abstract

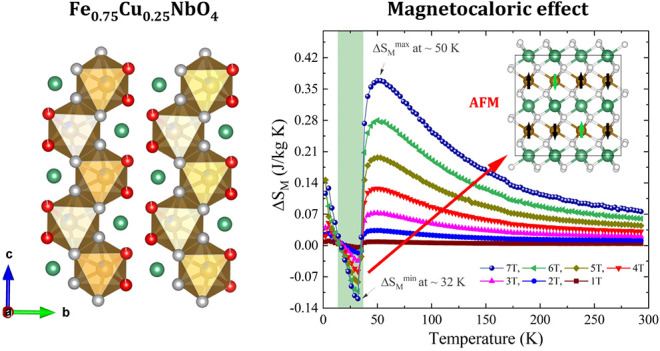

## 1 Introduction

The spin-chain magnetic behaviors of these systems combine geometric frustration with intrinsic low dimensionality, giving rise to complex physical phenomena that continue to attract great interest.^1–4^ Wolframite-type structures, specifically compounds with the general formula ABO_4_, where A = Fe, Ni and B = Nb, W,^5–7^ exhibit characteristics similar to 1D systems, with a zigzag spin-chain distribution. For example, the FeNbO_4_ (FNO) system, with monoclinic symmetry (m-FeNbO_4_) and space group *P*2/*c*, presents an ordered arrangement of Fe^3+^ and Nb^5+^ cations, octahedrally coordinated by six oxygen ions, forming zigzag chains composed of four octahedra in total for the Fe^3+^ and Nb^5+^ ions, denoted [FeO_6_] and [NbO_6_], along the *c*-axis [see Figure 1a].^8,9^ Its magnetic properties are predominantly governed by Fe^3+^ (3d^5^, s = 5/2) ions,^5^ where intrachain magnetic interactions (Fe–O–Fe) lead to ferromagnetic (FM) ordering, giving rise to infinite FM sheets aligned along the [100] direction.^10^ In contrast, interchain exchange interactions (Fe–O–Nb–O–Fe) induce AFM ordering below the Néel temperature (T_N_). Neutron diffraction, magnetization, and specific-heat studies indicate long-range magnetic ordering below T ≈ 38 K,^9,11–13^ with interchain interactions (Fe–O–Nb–O–Fe) driving AFM order below T_N_.

**Figure 1:**
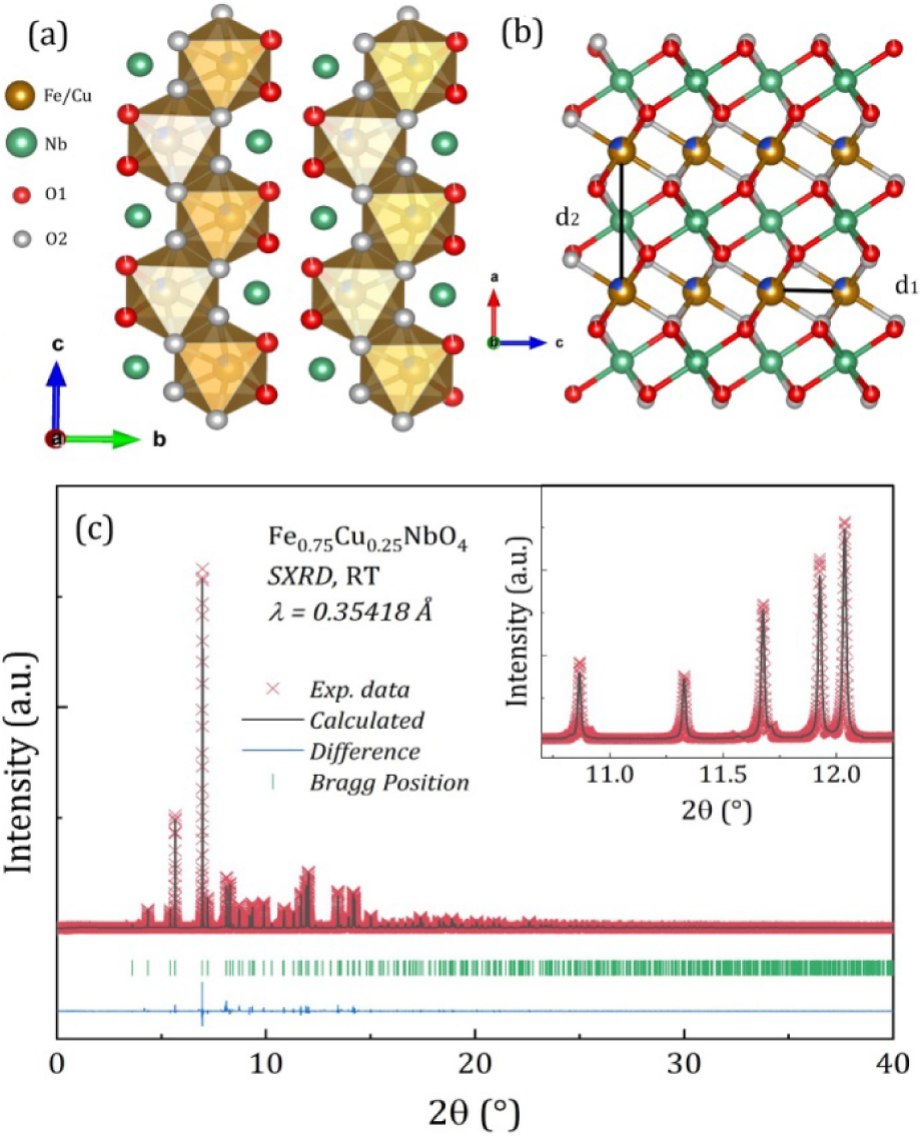
(a) Illustrative scheme of the FCNO crystal structure. (b) View along the *c*-axis showing the zigzag distribution of cations in the lattice and the distance between Fe–Fe (d_1_), Fe–Cu (d_1̀_) and Fe–Nb–Fe (d_2_). (c) SXRD Rietveld profile results at room temperature, where red crosses are the experimental data, black line is the fitted pattern, blue line denotes the difference between experimental and fitted patterns, and green vertical bars are the Bragg peak positions.

Recently, we showed that the ordering of FNO can be altered by partially replacing Fe^3+^ ions (s = 5/2) with Cu^2+^ ions (3d^9^, s = 1/2)^13^ In particular, a magnetic phase transition from AFM to FiM was demonstrated upon insertion of Cu^2+^ ions. Notably, substitution of Cu at concentrations higher than 5% promoted a change from global AFM ordering to FiM, with a critical ordering temperature (Tc) above 300 K. Similar magnetic-order transitions are observed in (Fe_1–*x*_Mn_*x*_)_2_Mo_3_O_8_ (*x* > 0.25)^14^ and (Mn_1–*x*_Co_*x*_)_2_Mo_3_O_8_ (0 ≤ *x* ≤ 1).^15^ However, deeper and more comprehensive investigations are imperative to fully understand the origin of the observed FiM order. This includes theoretical and experimental analyses that may clarify the contribution of induced defects to the magnetic properties of the Cu-substituted FNO structure. Understanding the structural and magnetic characteristics and the influence of defects in 1D structures are critical factors for explaining the intriguing magnetic behavior recently reported.^13^

Based on the features of the Cu-substituted FNO compounds, as well as their 1D-like magnetic character, we conducted a systematic experimental study on the Fe_0.75_Cu_0.25_NbO_4_ (FCNO) compound, probing its structural, vibrational, charge-state, and magnetic properties at low temperatures. Substituting one-quarter (25%) of Fe by Cu ions ensures a better ionic distribution in the octahedral configurations while preserving the strong magnetic character of the FNO compound established by Fe ions. We also note that substitution up to 15% Cu, which induces the AFM-to-FiM transition,^13^ requires further studies to understand, for example, the possible coexistence of AFM and FiM phases over a wide temperature range.

## 2 Experimental Section

### 2.1 Synthesis

The synthesis of the Fe_0.75_Cu_0.25_NbO_4_ (FCNO) sample was carried out using a modified solid-state reaction method. The procedure involved the precise measurement of the initial reagents, namely iron­(III) nitrate nonahydrate (Fe­(NO_3_)_3_·9H_2_O—Sigma-Aldrich, 98%), copper­(II) oxide (CuO—Sigma-Aldrich, 99%), and niobium­(V) pentoxide (Nb_2_O_5_ - Sigma-Aldrich, 99.9%), in stoichiometric quantities. Initially, Fe­(NO_3_)_3_·9H_2_O was dissolved in ethanol, and subsequently, the other reagents were introduced into the solution. The resulting mixture was subjected to drying at 80 °C for 1 h within a mini-muffle furnace, followed by sintering at 1000 °C for 6 h, all conducted under ambient atmospheric conditions.

### 2.2 Characterization

The high-resolution synchrotron X-ray diffraction (SXRD) powder pattern under ambient conditions was acquired at the European Synchrotron Radiation Facility (ESRF) beamline ID22 in Grenoble, France. The X-ray source operated at a wavelength (λ) of 0.35418 Å (35 keV), and the diffraction data were collected in a continuous scanning mode within the 2θ range of 1–40°. To minimize potential texture effects, the sample was securely enclosed within a quartz-glass capillary with a diameter of 0.5 mm and was measured under controlled rotation. Subsequently, the obtained SXRD pattern was subjected to a rigorous analysis through Rietveld refinement employing the *FullProf* program.^16^ This refinement procedure incorporated various parameters, including scale factors, zero-point error, background coefficients, asymmetry correction factors, lattice parameters, atomic positions, occupancy factors, and isotropic displacement parameters. Furthermore, the peak shape was characterized using a pseudo-Voigt function, and the background was interpolated between regions devoid of reflections. Low-temperature Raman spectroscopy was conducted employing a cryostat cell (THMS 600) equipped with precise temperature control capabilities. The Raman spectra were acquired in backscattering geometry, utilizing a Jobin Yvon T64000 triple-grating spectrometer configured in subtractive mode, with the slits adjusted to achieve a spectral resolution of 2 cm^–1^. The excitation source employed was an argon ion laser emitting at a wavelength of 514.5 nm. To focus the laser beam, an Olympus microscope lens with a focal distance (f) of 20.5 mm and a numerical aperture (NA) of 0.35 was employed. This setup ensured the generation of high-quality Raman spectra for the study. Magnetic property measurements were conducted employing a SQUID magnetometer, specifically the MPMS-3 system from Quantum Design (San Diego, USA). These measurements encompassed a low-temperature range spanning from 2 to 300 K and magnetic fields of up to 7 T. Transmission ^57^Fe Mössbauer spectra were obtained at 300 K and 15K using a conventional spectrometer equipped with a Janis He closed-cycle setup, with a sinusoidal velocity drive and a 25 mCi ^57^Co­(Rh) γ-ray source. The velocity scale of the spectra was calibrated relative to an α-Fe foil. Data processing was performed using the WinNormos software suite. X-ray photoelectron spectroscopy (XPS) measurements were performed using a Thermo Scientific K-alpha spectrometer equipped with a monochromatic Al–Kα source (photon energy = 1486 eV) at room temperature. Due to the insulating nature of the sample, charge compensation was implemented to ensure accurate spectral analysis. This was achieved by referencing the binding energy to the residual carbon peak, which was calibrated to the standard value of aliphatic carbon at 284.8 eV. This calibration allowed for the precise determination of binding energies across the sample.

## 3 Results

### 3.1 Synchrotron X-ray Diffraction

Room temperature Synchrotron X-ray Diffraction (SXRD), shown in Figure 1c, brought information on the structural properties of the FCNO powder sample. It was Rietveld refined unequivocally with the monoclinic phase that belongs to the space group *P*2/*c* (No. 13), in excellent agreement with the ICDD#00–070–2275 database. The quality metrics, namely χ^2^ = 21.7, *RF* = 3.55%, *R*_*Bragg*_ = 5.98%, and *R*_*p*_ = 4.62%, serve as indicators of the structural reliability. The unequivocal insertion of Cu^2+^ ions is observed at the Fe^3+^ sites (*2f* Wyckoff sites), as illustrated in Figure 1a and Figure S1a. Conventional Cu K_α_ laboratory XRD and Rietveld refinement (see Figure 1c, Table S1 in the Supporting Information) confirm the same single-phase FeNbO_4_ structure obtained by SXRD, with no detectable secondary phases. The derived lattice constants are *a* = 4.6567 Å, *b* = 5.6313 Å, and *c* = 5.0019 Å, which are also in good agreement with previous studies.^8–13^ In addition, a precise determination of cationic distances along the chains was obtained, namely *d*_1_ (Fe–Fe), *d’*_1_ (Cu–Fe), and *d*_2_ (Fe–Nb–Fe), revealing that *d*_1_ = *d’*_1_ (∼3.17 Å) and *d*_2_ = 4.65 Å, as depicted in Figure 1 b. The results of the Rietveld refinement are presented in detail in Table 1.

**Table 1:** Structural Parameters at Room Temperature and Quality Factors

Crystallographic data (space-group: P21/c)							
Atom	Wyck. Posit.	x	y	z	Occ.	B	Mult.
Fe	2*f*	0.5000(0)	0.6703(8)	0.2500(0)	0.379(0)	0.524(1)	2
Cu	2*f*	0.5000(0)	0.6703(8)	0.2500(0)	0.129(0)	0.524(1)	2
Nb	2*e*	0.0000(0)	0.1807(4)	0.2500(0)	0.500(0)	0.520(1)	2
O1	4*g*	0.2313(5)	0.1149(1)	0.5812(3)	0.969(8)	0.843(9)	4
O2	4*g*	0.2697(9)	0.3838(5)	0.0831(9)	0.973(7)	0.287(8)	4

Lattice Parameters			
a = 4.6567(9) (Å)	b = 5.6313(7) (Å)	c = 5.0019(1) (Å)	V = 131.22(6) (Å^3^)
α = 90.00(°)	β = 90.01 (°)	γ = 90.00 (°)	ρ = 5.379(g/cm^3^)
Discrepancy factors: χ^2^ = 21.7, *R*F = 3.55*%, R*_*Bragg*_ *= 5.98%, and R*_*exp*_ *= 4.62%*			

Lengths and Angles bond (Å)					
Fe–O1 (Å)	Fe–O2 (Å)	Nb–O1 (Å)	Nb–O2 (Å)	Fe–O2–Fe	Fe–O2–Nb
1.934(4)	2.006(4)	2.011(4)	1.893(4)	100.0	128.6(8)
	2.109(4)	2.156(4)			

### 3.2 X-ray Photoelectron Spectroscopy (XPS)

The stoichiometry of the surface and the chemical states of elements in the Cu-substituted FeNbO_4_ sample were assessed by analyzing the Fe-2*p*, Cu-2*p*, Nb-3*d*, and O-1*s* core levels by XPS, as shown in Figure 2. The Nb-3*d* core-level spectrum (Figure 2a) was deconvoluted into two peaks corresponding to Nb-3*d*_5/2_ and its spin–orbit counterpart Nb-3*d*_3/2_, with a spin–orbit splitting of 2.75 eV. The binding energies of the doublet peaks, Nb-3*d*_5/2_ and Nb-3*d*_3/2_, were found to be 206.2 and 209.1 eV, respectively, and they are consistent with the reported peak positions for Nb^5+^ ions in the Nb_2_O_5_ structure.^17–19^ The Fe-2*p* core-level spectrum, shown in Figure 2b, reveals two primary peaks at 710.2 eV (Fe-2*p*_3/2_) and 724.6 eV (Fe-2*p*_1/2_), both characteristic of Fe^3+^ ions in an octahedral coordination, as would be expected for this compound. The presence of broad satellite peaks at approximately 718.9 and 731.8 eV further confirms the Fe^3+^ oxidation state.^17,20^ Therefore, the Nb and Fe XPS results first indicate that the Cu-substitution does not alter the Fe^3+^ states in the FeNbO_4_ sample, but they also suggest that the octahedral coordination for Fe is kept unchanged.

**Figure 2:**
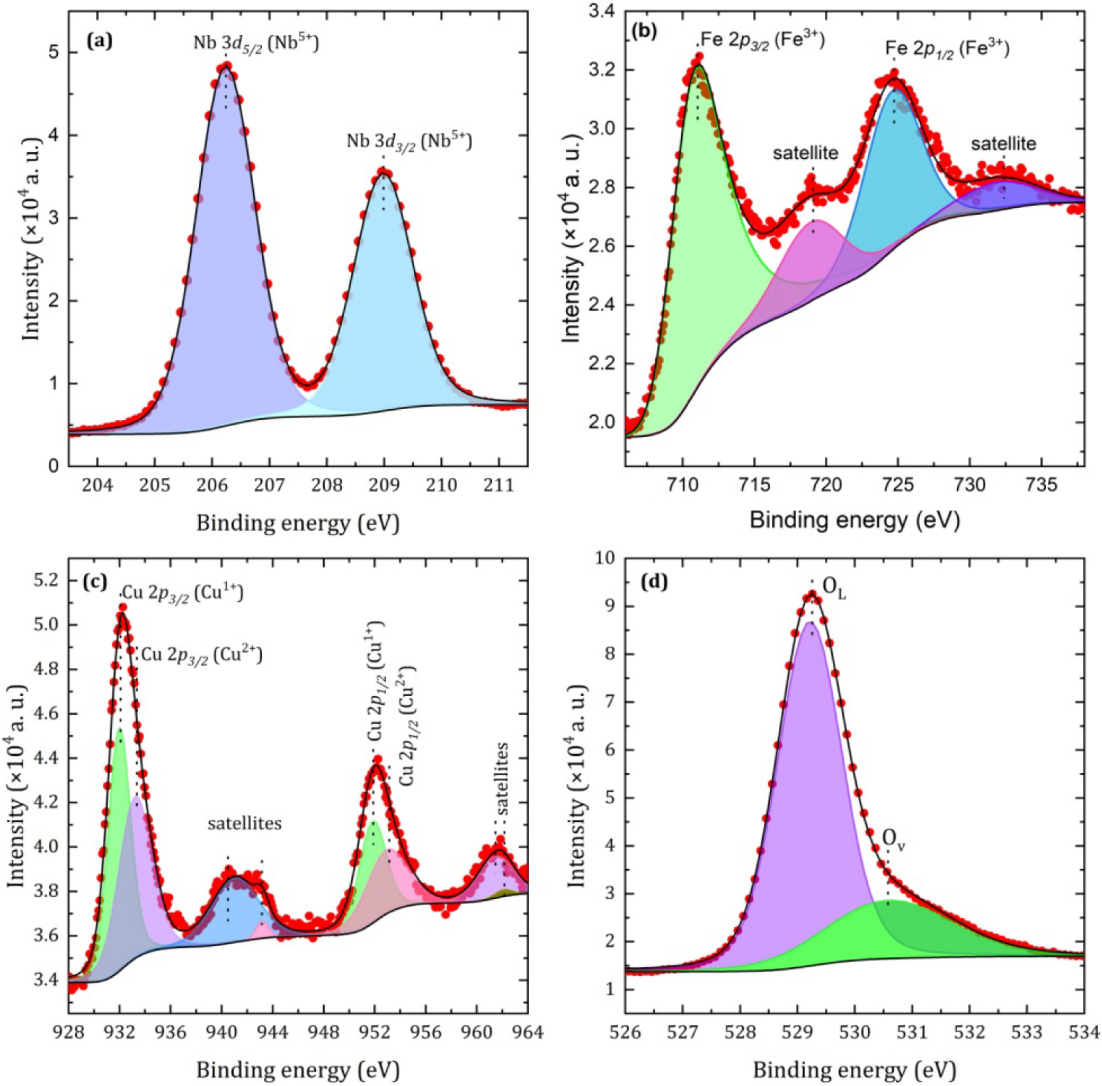
High-resolution Nb-3*d* (a), Fe-2*p* (b), Cu-2*p* (c), and O-1*s* (d) XPS spectra for the Cu-doped FeNbO_4_ sample.

The deconvoluted high-resolution XPS spectra of the Cu-2*p* core levels are presented in Figure 2c. Two subpeaks were observed for both Cu-2*p*_3/2_ and Cu-2*p*_1/2_, with binding energies of 933.5 and 955.5 eV, respectively. The binding energy separation of 19.9 eV is indicative of the Cu^2+^ oxidation state.^21,22^ This assignment is corroborated by the appearance of four satellite peaks at 940.9, 942.8, 960.9, and 962.3 eV, which are attributed to the “shake-up” processes involving excitation of electrons to higher energy states.^23–26^ In addition, Cu-2*p*_3/2_ and Cu-2*p*_1/2_ peaks at 931.7 and 951.4 eV, respectively, suggest the presence of Cu^1+^ and/or Cu^0^ due to their overlapping binding energies. To distinguish these oxidation states, Cu LMM Auger peak analysis was performed. As displayed in Figure. S2, a narrow Cu LMM peak at 568.6 eV corresponds to Cu^2+^, whereas a peak at 571.1 eV corresponds to Cu^1+^ rather than Cu^0^, which typically appears at 568 eV.^27,28^ The O-1*s* XPS spectrum, shown in Figure 2d, was fitted to two distinct peaks corresponding to lattice oxygen (O_L_) at 529.2 eV and oxygen vacancies (O_v_) at 530.5 eV.^17,29^

### 3.3 ^57^Fe Mössbauer Spectroscopy

Further evidence for Cu-induced defects or local disorder in the FeNbO_4_ is provided by temperature-dependent ^57^Fe Mössbauer spectroscopy. As shown in Figure 3, the Mössbauer spectra for FCNO show clearly two Fe-species for both temperatures, i.e., at 300 and 15 K. A paramagnetic component (a slightly asymmetric doublet in the central part of the spectrum) and a magnetic subspectrum (sextet) form the 300 K spectrum, while at 15 K both Fe-species are in a magnetically ordered state. Consequently, these spectra were fitted with two subspectra to account, at least two different Fe-species, namely Fe–I and Fe–II. The fitted Mössbauer parameters can be seen in Table 2.

**Figure 3:**
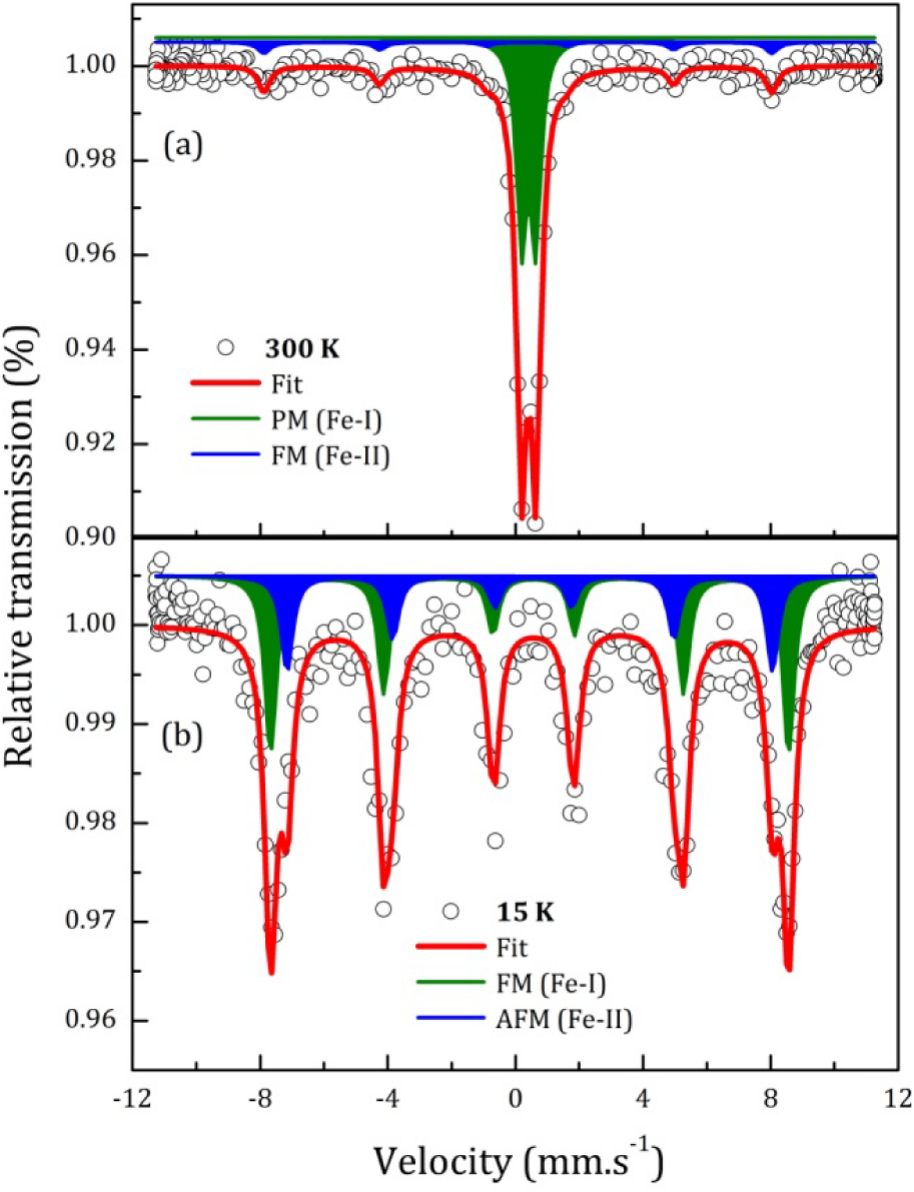
^57^Fe Mössbauer spectroscopy spectra of Cu-doped FeNbO_4_ sample measured at 300 K (a) and 15 K (b).

**Table 2:** Mössbauer Fitted Parameters^a^

300 K					
Fitting	B_hf_(*T)*	δ (mm s^–1^)	QS (mm s^–1^)	ε (mm s^–1^)	f (%)
**Doublet (Olive)**		0.42	0.45		85
**Sextet (Blue)**	49.00	0.22		–0.27	14

15 K					
**Sextet (Olive)**	50.2(2)	0.51(1)		–0.13	67(2)
**Sextet (Blue)**	47.2(5)	0.51(1)		0.10	33(2)

a B_*hf*_ hyperfine magnetic field, isomer shift, *Q.S*. quadrupole splitting, *ε* hyperfine splitting, *f* relative area.

At 300 K, a doublet with isomer shift (δ) equal to 0.42 mm/s, quadrupole splitting (QS) of 0.45 mm/s, and fraction (*f*) of 83% and a sextet with δ = 0.22 mm/s, quadrupole splitting (ε) of −0.27 mm/s, magnetic hyperfine field (*B*_*hf*_) of 49 T, and *f* = 17%. At 15 K, two sextets with similar δ of 0.51 mm/s (here the second order Doppler shift is present due to sample and source at different temperatures^30^), but with *ε* = −0.13 mm/s, *B*_*hf*_ = 50.2 T and *f* = 67% for the Fe–I species and *ε* = 0.10 mm/s, *B*_*hf*_ = 47.2 T and *f-* = 33% for the Fe–II species. The identical δ values confirm only the presence of the Fe^3+^ state.^31,32^

### 3.4 Evaluating Magnetic Order Transitions

The isotherm M-H curves obtained at temperatures of 1.8, 60, 80, and 300 K (as depicted in Figure 4a) clearly manifest a substantial enhancement in magnetization as temperature is decreased. Specifically, the highlight in Figure 4a emphasizes the behavior of these curves in the low-field region, unveiling coercive field (H_C_) and remanent magnetization (M_r_) values of approximately H_C_ ∼ 0.018 T and M_r_ ∼ 0.72 emu/g. The presence of hysteresis and low magnitudes of H_C_ and M_r_ corroborate the manifestation of FiM order in the FCNO sample in all temperature ranges (the sextet at 300 K also supports this observation). For example, 300 K M-H curve may be fitted with two main contributions: a FiM order due to the loop hysteric effect and a paramagnetic (PM) component that does not allow the sample magnetization saturation, similar to the 300 K Mössbauer result [doublet (PM) plus sextet (FiM)].

**Figure 4:**
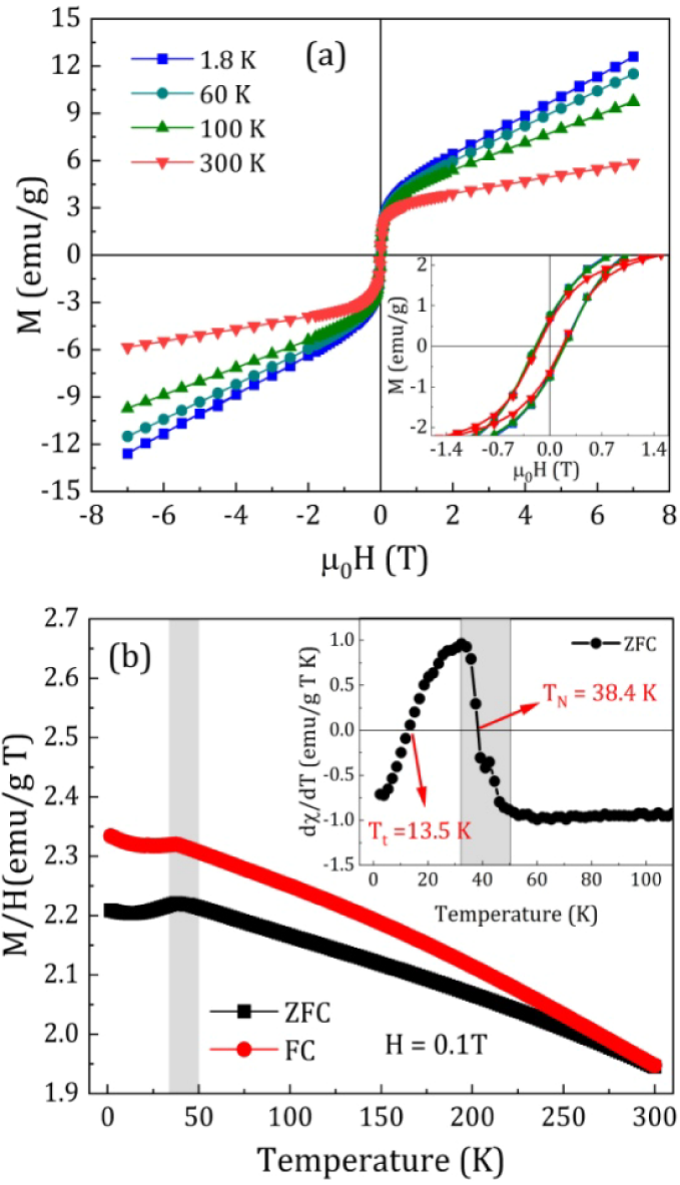
(a) *M-H* curves measured at different temperatures (*T* = 1.8, 60, 100, and 300 K), the inset exhibits the magnification of the low-field region.(b)­Magnetic susceptibility (χ = *M*/*H*) measurements as a function of temperature (*T*) on the zero-field cooling (ZFC) and field cooling (FC) protocols for the FCNO. In the inset, the derivative of the magnetic susceptibility (*dχ*/*dT*) indicates the transition regions.

The observation that the saturation magnetization (M_S_), M_r_, and H_C_ display minimal variations across this temperature range is also a behavior uncommon in ordinary magnets; consequently, it warrants further investigation. The magnetic susceptibility (χ_*DC*_ *= M/H*) as a function of temperature (T), in the zero-field cooling (ZFC) and field-cooling (FC) protocols for FCNO, is depicted in Figure 4b. First, the ZFC and FC curves are not reversible (split) already at high temperatures (<275 K), evidencing a magnetically ordered state as that observed in the 300 K Mössbauer spectrum. However, it should be mentioned that the magnetization experiences a gradual increase as the sample temperature decreases up to approximately 50 K, when both the ZFC and FC curves manifest a descending magnetization tail. This observation is indicative of a typical low-temperature AFM ordering behavior, probably due to those Fe-species that were in PM state, as indicated by 83% of the 300 K spectrum (Figure 3). First, at approximately 32 K, a broad peak is observed in the χ_*DC*_ curve. It can be attributed to regions of the sample where long-range interactions are more effectively established, signifying AFM state expected for the sample matrix, i.e., a matrix with the pristine FNO structure. However, it is also relevant to observe that the susceptibility derivative (*dχ/dT*) presents two inflection points at T_t_ = 13.5 K and T_N_ = 38.4 K, as illustrated in the inset of Figure 4b, indicating two magnetic transition temperatures.

### 3.5 Magnetocaloric Effect

As the magnetic entropy is inherently sensitive to small fluctuations in magnetization, making it an invaluable resource for studying magnetic states and phase transitions,^1^ we turned our attention to the magnetocaloric effect (MCE), analyzing the variation of magnetic entropy (*ΔS*_*M*_) as a function of temperature under various applied magnetic fields (μ_0_H), to further unraveling the AFM-FiM magnetic features found in the FCNO system. It is worth noting that, to the best of our knowledge, the MCE has not been previously documented for this specific sample. For this, M-H curves were acquired over the temperature range of 2–300 K, with temperature intervals (*ΔT*) set at 3 K, and under magnetic fields (μ_0_ *H*) up to 7 T, as presented in Figure 5a. Notably, the M-H isotherms reveal a less steep increase in magnetization with rising temperature. Subsequently, these isotherms were transformed into Arrott plots (H/M vs M^2^),^33^ as depicted in the inset of Figure 5a. The obtained Arrott slopes for all curves are positive, indicating the presence of a second-order magnetic phase transition (SOPT) for the FCNO sample. From the M-H curves, *ΔS*_*M*_ *(T)* was estimated using the following relation:

**Figure 5:**
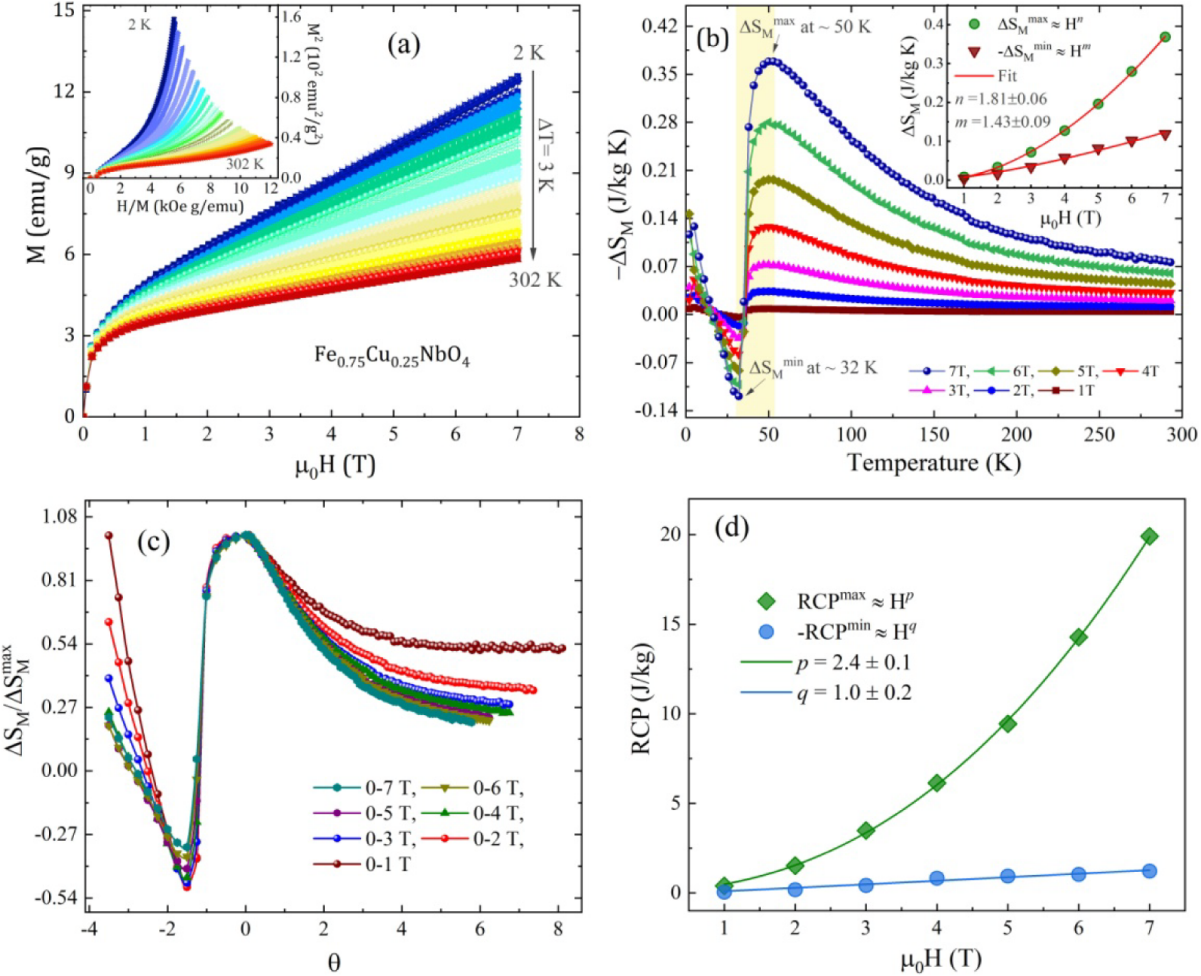
(a) Isothermals *M-H* field-dependent up to 7 T measured from *T* = 2 to 302 K for the FCNO sample. The inset shows the Arrott plots (*H*/*M vs M* ^2^). (b) Temperature-dependent *ΔS*_*M*_ at various magnetic fields changes from 1 up to 7 T. The inset demonstrates the power-law dependence of the $\Delta S_{M}^{\max}$ ­(*H*) and $\Delta S_{M}^{\min}$ ­(*H*) curves. (c) $\Delta S_{M}/\Delta S_{M}^{\max}$ vs θ curves. (d) Magnetic field-dependent *RCP* ^*max*^ and *RCP* ^*min*^ curves.

1 $$\Delta S_{M}\left(T,H_{0}\right)=\underset{i}{\sum }\frac{M_{i+1}-M_{i}}{T_{i+1}-T_{i}}\Delta H_{i}$$

where *M*_*i*_ and *M*_*i+1*_ denote the experimental data obtained at temperatures *T*_*i*_ and *T*_*i+1*_ under a magnetic field strength *H*_*i*_, respectively.^34^ In Figure 5b, the *ΔS*_*M*_ *(T)* profiles are displayed at various magnetic fields ranging from 1 to 7 T for FCNO. These profiles exhibit a positive broad *ΔS*_*M*_ peak ca. 50 K, and with $\Delta S_{M}^{max}$ value progressively increases with the μ_0_ *H*. This behavior is a well-established characteristic of the conventional MCE and arises from the resultant FiM interactions within the sample. Subsequently, an inversion from positive to negative values is observed at approximately 36 K (defined as T_N_), with $\Delta S_{M}^{min}$ ≈ 32 K (inverse MCE), which corresponds to the well-known transition from a conventional MCE (at *T* > T_N_) to an inverse MCE (at *T* < T_N_).^35,36^ These results may indicate a temperature-dependent magnetic phase transition region (FiM-AFM).

The values of $\Delta S_{M}^{max}$ and $-\Delta S_{M}^{min}$ were found to exhibit a power-law dependence on μ_0_ *H*, such that $\Delta S_{M}^{max}\approx H^{n}$ and $-\Delta S_{M}^{min}\approx H^{m}$ , as illustrated in the inset of Figure 5b. Notably, the obtained exponents are *n* = (1.81 ± 0.06) and *m* = (1.43 ± 0.09), respectively, where *n* deviates from the ideal mean-field FM value of *n* = 2/3.^37,38^ This departure from the mean-field value is indicative of possible influences stemming from the presence of magnetic domains in the vicinity of the transition temperature. Furthermore, the substantial increase in Δ*S_M_* (*H*) underscores the potential for enhanced magnetocaloric effects, which can be further fine-tuned through adjustments in the applied magnetic field intensity.

Then, we conducted the AFM-FiM magnetic order transition through the rescaling of Δ*S_M_* curves $\left(\Delta S_{M}/\Delta S_{M}^{max}\right)$ , parametrized by the reduced temperature θ _±_ , as expressed by the following equations:

2 $$\theta_{-}=\left(T_{\mathrm{peak}}-T\right)/\left(T_{r1}-T_{\mathrm{peak}}\right),\;T<T_{\mathrm{peak}}$$

3 $$\theta_{+}=\left(T-T_{\mathrm{peak}}\right)/\left(T_{r2}-T_{\mathrm{peak}}\right),\;T>T_{\mathrm{peak}}$$

Here, *T_r_*_1_ and *T_r_*_2_ denote the temperatures of two reference points determined by $\Delta S_{M}\left(T_{r1},T_{r2}\right)=\frac{1}{2}\Delta S_{M}^{\max}$ .^39^ In Figure 5(c), the $\Delta S_{M}/\Delta S_{M}^{\max}\left(\theta \right)$ curves are presented for magnetic field strengths ranging from μ_0_ *H* = 0 to 7 T, revealing the presence of two distinctive minima and maxima. The maximum peak converges into a single curve in the vicinity of the peak (θ ≈ 0), while the minimum peak (θ ≈ −1.5) does not exhibit a similar convergence. This observation corroborates the AFM-FiM competing interactions.^13^

In connection with the preceding findings, the evaluation of the relative cooling parameter (RCP) for the FCNO sample was undertaken, employing the expression $\mathrm{RCP}=\left|\Delta S_{M}^{\max}\right|x\delta T_{\mathrm{FWHM}}$ , where *δT*_FWHM_ represents the full width at half-maximum of the *ΔS*_*M*_ *(T)* curves.^40^ Figure 5(d) presents the *RCP* ^*max*^ and *RCP* ^*min*^ profiles as functions of the magnetic field derived from the $\Delta S_{M}^{\max}$ ­(*H*) and $\Delta S_{M}^{\min}$ ­(*H*) curves, respectively. At μ_0_ *H* = 7 T, the maximum values are RCP^max^ = 19.9 J/kg and RCP^min^ = 1.2 J/kg. Both RCP^max^ and RCP^min^ exhibit power-law dependencies on the magnetic field, expressed as RCP^max^ ≈ *H^P^* and RCP^min^ *≈ H^q^*, yielding *p* = (2.4 ± 0.1) and *q* = (1.0 ± 0.2). It is worth noting that the *p*-value signifies a robust dependence of RCP on the magnetic field, which can be attributed to the broadening of peaks in the magnetic-phase transition region related to $\left|\Delta S_{M}^{\max}\right|$ . In contrast, the *q*-value displays an almost linear increase. However, these determined exponents are smaller than those observed in analogous materials, such as RVO_4_(R = Gd, Nd, Ho, Er, and Yb).^41,41–43^ This deviation may be attributed to long-range magnetically disordered clusters in FCNO, resulting in a diminished magnetic entropy change.

### 3.6 Raman Spectroscopy

Raman spectroscopy was performed at different temperatures to understand the phonon behavior through the magnetic anomalies seen in the FCNO susceptibility curves. Raman spectra were recorded from 10 to 300 K, as shown in Figure 6a. We analyzed the Raman modes using a Lorentzian function, shown in detail in Figure S3. The identification and assignments of each vibration mode are shown in Table S2. The Raman modes generally shift to low frequency as the temperature increases, accompanied by a monotonic increase in fwhm.^44^ This is mainly due to the expansion of the lattice as the thermal energy increases. The absence of any extra peaks indicates that the spectral symmetry remains the same at all measured temperatures, i.e., the FCNO sample does not show a structural phase transition in this temperature range.

**Figure 6:**
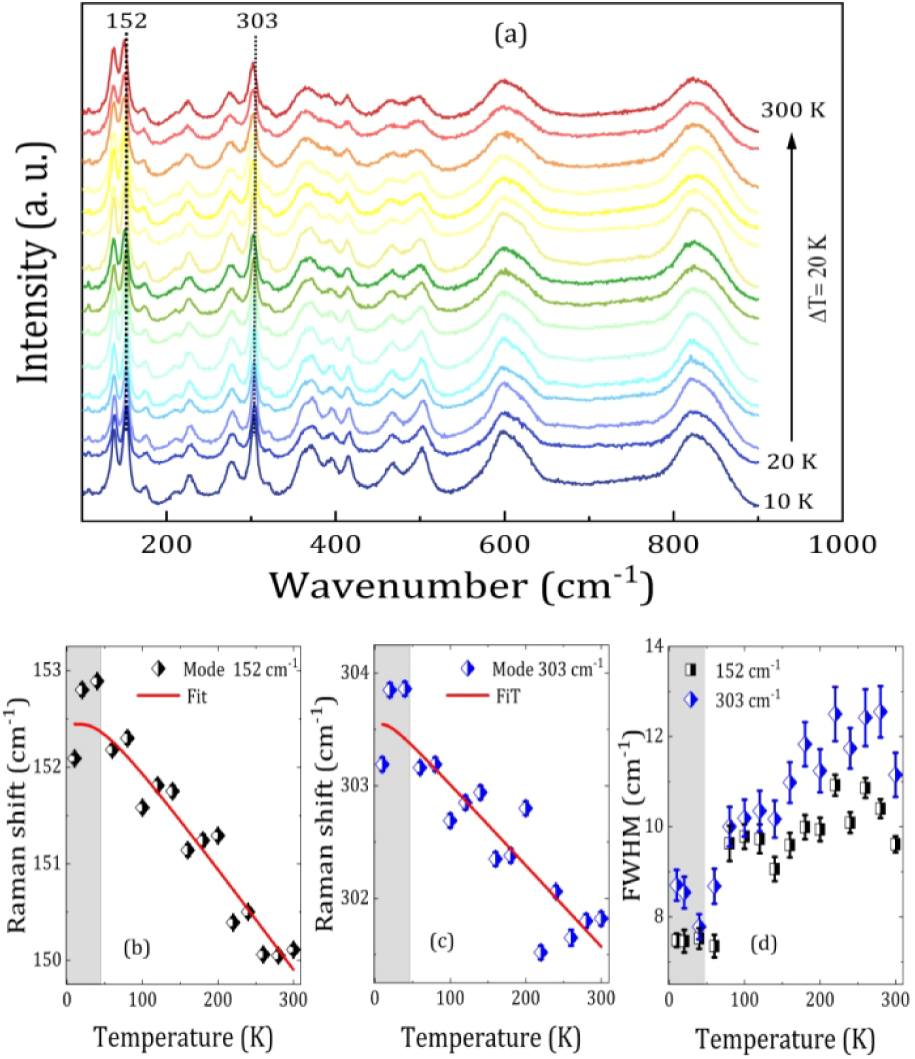
(a) Raman spectra of FCNO as a function of temperature from 10 to 300 K. Raman shifts as a function of temperature for localized modes in (c) 152 cm^–1^, (d) 303 cm^–1^.

The temperature dependence of the phonon frequencies of the 152 cm^–1^ and 303 cm^–1^ modes, together with the fit assuming a standard anharmonic dependence of the phonon modes, is shown in Figure 6a–c. The experimental data were fitted to the Balkanski model, which describes the position of a phonon as temperature dependent, where only the anharmonic contributions of the modes as a function of temperature are considered, given by the following equation:^44,45^

4 $$\omega \left(T\right)=\omega_{0}-C_{1}\left(1+\frac{2}{e^{\hbar \omega_{0}/2k_{B}T}-1}\right)+C_{2}\left[1+\frac{3}{e^{\hbar \omega_{0}/3k_{B}T}-1}+\frac{3}{{\left(e^{\hbar \omega_{0}/3k_{B}T}-1\right)}^{2}}\right]$$

where ω_0_ is the temperature-independent part of the line width, C_1_ and C_2_ are adjustable parameters, where the terms multiplied by C_1_ are contributions due to the 3-phonon decay process and the terms multiplied by C_2_ are contributions due to the 4-phonon decay process, *ℏω*_0_ is the phonon energy, and *k*_*B*_ is the Boltzmann constant.^46^ The deviation in the phonon frequency from the anharmonic dependence near T_N_ can be clearly seen in Figure 6a-b. In Figure 6c, between 260 and 300 K or below 100 K, the fwhm shows a significant reduction, which can be associated with magnetic transition temperatures, i.e., it is indicative of a magnetic phase transition followed by a spin-phonon coupling observed by temperature-dependent Raman spectroscopy.^44,45,47^ According to Granado et al., the anomalies observed in the frequencies of the modes, when caused by spin-phonon coupling, can be described by the following equation:^48^

5 $$\Delta \omega_{S-ph}\left(T\right)=\lambda \left\langle S_{i}\cdot S_{j}\right\rangle \approx \lambda {[\chi \left(T\right)/\chi_{\max}]}^{2}$$

Here, Δ*ω_S_*_‑_ *_ph_* (*T*) = (*ω_obs_* - *ω*_0_), where ω_obs_ is the observed phonon frequency, ω_0_ denotes the phonon frequency in the absence of spin correlations, λ is the spin-phonon coupling constant, ⟨*S_i_* . *S_j_* ⟩ is the spin–spin correlation function of adjacent spins, and χ­(T) is the magnetic susceptibility^49^

Figure 7 (a-b) shows the phonon shift (Δω) and squared normalized magnetic susceptibility $\left({[\chi \left(T\right)/\chi_{\max}]}^{2}\right)$ data as a function of temperature, where we can see that for both modes there is good agreement with eq 5). Both modes exhibit clear anomalies at T_N_ ≃ 38.4 K (kinks in ω­(T) and Γ­(T), extreme in Δω_s‑ph_(T), consistent with AFM spin–phonon coupling.^49^ Thus, these results also support the two magnetic states observed by Mössbauer and magnetization experiments, with magnetic transitions not associated with structural phase transformation, but due to Fe^3+^ interactions at different environments, as previously discussed.

**Figure 7:**
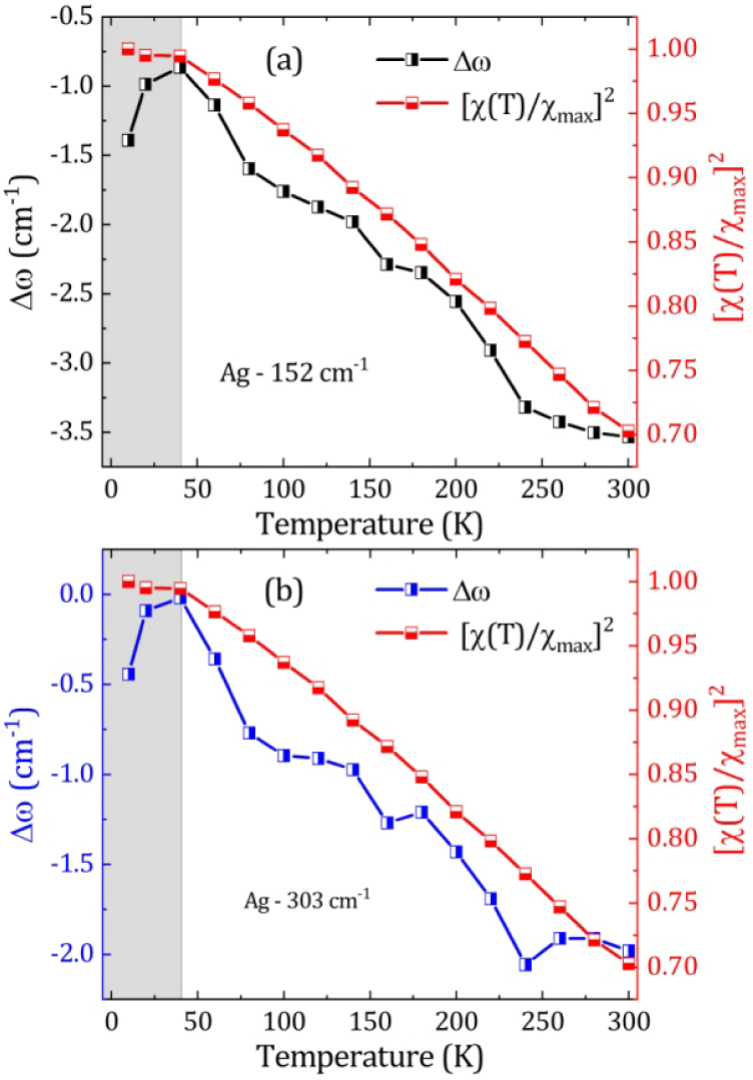
Phonon shift (*ω_obs_* - *ω*_0_) and squared normalized magnetic susceptibility ([*X*(*T*)/*X_max_*]^2^) as a function of temperature for the 152 cm^–1^ (a) and 303 cm^–1^ (b) modes.

## 4 Discussion

We present multiple experimental observations demonstrating that the FCNO compound undergoes two temperature-dependent magnetic transitions at T_t_ = 13.5 and T_N_ = 38.4 K, consistent with a FiM order. To contextualize these phenomena, we first consider the magnetic behavior of the pure sample. FeNbO_4_ is a characteristic AFM with an ordering temperature of ∼ 38.5 K in which Fe^3+^ ions adopt a high-spin configuration with S = 5/2. Interchain exchange interactions between Fe^3+^-Fe^3+^ are presumably FM, whereas the dominant intrachain (Fe^3+^–Nb-Fe^3+^) interactions are AFM within the ordered state.^11–13^ For instance, recent DFT studies^8^ report two additional low-energy magnetic configurations for FeNbO_4_, implying near degeneracy among three magnetic motifs and the possibility of domain-selective coexistence. Such energetic proximity suggests that modest perturbations can tip the balance among competing orders. In our case, substituting Fe^3+^ with Cu^2+^ perturbs the parent antiferromagnet via spin dilution and local structural/electronic distortions associated with Cu^2+^ (d^9^), which modify superexchange pathways and the relative strengths of interchain and intrachain interactions. Consistent with this picture, an AFM-to-FiM transformation emerges in Fe_1–x_Cu_*x*_NbO_4_ for *x* > 0.05, indicating that Cu incorporation stabilizes a ferrimagnetic arrangement that is otherwise only a low-lying alternative in the undoped compound.^13^

Furthermore, because FCNO exhibits two temperature-driven magnetic transitions, we first assess how replacing 25% of Fe^3+^ (3d^5^, S = 5/2) with Cu^2+^ (3d^9^, S = 1/2) alters the crystal structure. Rietveld refinement of the SXRD data shows that Cu incorporation modifies the lattice parameters and unit-cell volume, and introduces measurable changes in the local coordination. In particular, the insertion of 25% Cu induces significant changes in the structural parameters of the FCNO crystal structure. Notable variations are discerned, including an expansion in the lattice parameters (a, b, c), an increase in the unit cell volume, and a reduction in the β angle. These changes can be attributed to subtle local perturbations, such as an elongation in the O2-(Cu/Fe)-O2 bond lengths and (Fe/Cu)-O2-(Fe/Cu) bond angles [see Figure S1b and Table 1], compared to the reference material FNO.^13^ Consequently, the intrachain distance d_1_ increases from 3.13 to 3.17 Å, while d_2_ remains unchanged at 4.65 Å. These localized changes give rise to an increase in the (Fe/Cu)–O2–(Fe/Cu) bond angles, thus amplifying the distortion within the octahedral lattice (Figure S1). This increase can be explained by the difference in ionic radius of Cu^2+^ that has 0.730 Å in octahedral coordination, while Fe^3+^ has 0.645 Å, as well as by the strong Jahn–Teller effect^50^ caused by the Cu^2+^(3d^9^) ion, see Figure S1b. The increase in local disorder caused by the Cu insertion is also verified by Raman spectroscopy.^13^ The introduction of 25% Cu^2+^ ions gives rise to localized atomic perturbations at each lattice site, which in turn manifest as an increase in the vibrational frequencies. For example, the Raman shift for the Ag mode, related to the symmetric stretching of NbO_6_ plus a bending of FeO_6_,^51^ is observed at 818 cm^–1^ for FeNbO_4_,^13^ while for FCNO, it is recorded at 827 cm^–1^ (see Figures S3 and 6a), representing a Δũ of 9 cm^–1^. The occurrence of this Raman shift, together with an increase in the full width at half-maximum (fwhm),^13^ conclusively substantiates the claim that Cu^2+^ promotes cationic disorder within the monoclinic structure, directly influencing the magnetic ordering state of FCNO.^13,52^

Another essential analysis to elucidate the magnetic properties of the FCNO compound is to identify the chemical state of the element present in the sample. The surface stoichiometry and chemical states of the elements in the FCNO sample were determined by XPS, as shown in Figure 2. The results for Nb *3d* and Fe *2p* [Figure 2 a-b] first indicate that Cu insertion does not alter the states of Nb^5+^ and Fe^3+^ in the FCNO sample. The high-resolution XPS spectra of the Cu *2p* core levels [Figure 2c] together with the Auger peak analysis of Cu LMM (Figure S2), identify two subpeaks observed for Cu *2p*_*3/2*_ and Cu *2p*_*1/2*_, which are attributed to the presence of Cu in two oxidation states, Cu^1+^ and Cu^2+^. The observed Cu^1+^/Cu^2+^ ratio of 0.8 indicates a slight predominance of Cu^2+^, which plays a crucial role in stabilizing the material by maintaining charge neutrality and minimizing lattice distortions. The coexistence of Cu oxidation states creates a balanced electronic environment, reducing structural strain and increasing the overall stability of the material. The presence of oxygen vacancies [Figure 2d] further reinforces the role of Cu^2+^ in stabilizing the lattice structure by compensating for local charge imbalances. Notably, the percentage of *O*_*v*_ */(O*_*L*_ *+O*_*v*_) was 1/4, suggesting that Cu insertion induced the formation of oxygen vacancies. Furthermore, the atomic percentages of Cu, Fe, Nb, and O were approximately 5.6%, 9.0%, 16.0%, and 52.2%, respectively, closely aligning with the 1:1:4 atomic ratio (Cu/Fe):Nb:O of FeNbO_4_, providing strong evidence for the stabilization of the FeNbO_4_ phase in the Cu-doped system.

Evidence for Cu-induced defects or local disorder in FCNO is provided by the temperature-dependent Mössbauer spectroscopy of ^57^Fe, Figure 3. The Mössbauer spectra obtained at different temperatures confirm the influence of Cu on the local magnetic ordering of the FCNO sample. At 300 K, two subspectra arising from two ordered states of Fe^3+^ in FCNO are observed. The major PM component (doublet) is characteristic of the FNO compound.^53^ However, the observed sextet is clearly a localized FM ordered state of Fe^3+^ influenced by the presence of Cu^2+^ as first neighbors, Fe^3+^-Cu^2+^. This result confirms the global FiM ordered state of FCNO, when we take into account the Fe^3+^-Cu^2+^ interactions, since the Mössbauer spectra show only Fe–Fe interactions. For example, 300 K M-H curves [Figure 4a] can be fitted with two main contributions: an FiM order due to the loop hysteretic effect and a PM component that does not allow the sample magnetization to saturate. This result agrees with that for the 300 K Mössbauer spectrum [doublet (PM) plus sextet (FM)].

The spectrum measured at 15 K [Figure 3b] further highlights the local influence caused by Cu insertion. The differences in the hyperfine parameters ε and *B*_*hf*_ between the Fe–I and Fe–II species indicate distinct local Fe environments that require further explanation. The FM-ordered Fe–I species (initially magnetically disordered at 300 K) can be associated with temperature-induced effects in the Fe sublattice, largely unaffected by Cu ions. In contrast, the AFM-ordered Fe–II species (initially FM-ordered at 300 K) can be interpreted as arising from the AFM ordering of Fe^3+^ influenced by the incorporation of Cu^1+^ and Cu^2+^ ions. The mixed valence state of Cu introduces local distortions into the octahedral environment, primarily through Jahn–Teller effects associated with (Cu^2+^, t_2g_ ↑),^50^ which modify the electric field gradient (EFG) and result in the observed differences in quadrupole shift and hyperfine fields found in the Fe–I and Fe–II species. Notably, oxygen vacancies also play a significant role in influencing the hyperfine parameters. These vacancies disrupt the local electronic environment around the Fe ions, altering the EFG and contributing significantly to variations in *B*_*hf*_ and ε between the Fe–I and Fe–II species, as observed experimentally. Specifically, oxygen vacancies reduce the symmetry of the FeO_6_ octahedra, increasing the anisotropy in the hyperfine magnetic field (*B*_*hf*_) and impacting the quadrupole shift (ε). Thus, such vacancies may also indirectly affect magnetic exchange interactions by modifying the Fe^3+^–Fe^3+^ and Fe^3+^–Cu^2+^ superexchange pathways, partially explaining the differences in magnetic ordering between Fe species. Therefore, the distinct *B*_*hf*_ values for Fe–I and Fe–II species suggest differences in the orientation of the hyperfine field (hence the Fe magnetic moment) with respect to the main axis of the EFG, probably aligned with the *c* axis of the crystal. Anisotropic contributions to *B*_*hf*_ in Fe ions may arise from the influence of neighboring Cu^2+^ and oxygen vacancies, which alter the magnetic anisotropy of the lattice. Although Cu^1+^ does not directly participate in magnetic exchange, it modifies the electron density and bond geometry, indirectly impacting magnetic ordering.

The analysis of the results of the temperature-dependent magnetization curves clearly shows two magnetic phase transition temperatures present in FCNO, the inset of Figure 4a. The derivative of the magnetic susceptibility with respect to temperature points to two temperatures at T_N_ = 38.4 K and T_t_ = 13.5 K. The first transition temperature T_N_ is defined as the transition temperature from FiM to AFM, as the temperature is reduced. Whereas, T_t_ is defined as a transition temperature from AFM to FiM, i.e., a temperature of return to the FiM state. The value of T_N_ and AFM ordering state is similar to that observed for the pure sample. The transition observed at 13.5 K [Figure 4b] is somewhat complex and worth discussing. In FeNbO_4_, the increase in magnetization for *T* < T_N_ is generally attributed to short-range spin ordering with a small fraction of Fe^3+^ in the Nb chains.^11,54^ However, the Mössbauer spectrum obtained at 15 K [Figure 3a] can shed light on this phenomenon. Thus, T_t_ = 13.5 K can be attributed to a spin reorientation process in the Fe sublattice. Meanwhile, Fe^3+^ in the Fe/Cu sublattice is coupled by AFM. Therefore, the increase in short-range interactions between Fe^3+^-Fe^3+^ is mainly responsible for the reestablishment of the FiM order below T_N_.

Magnetic entropy is inherently sensitive to small fluctuations in magnetization, making it an invaluable resource for studying magnetic states and phase transitions.^1^ Therefore, we turned our attention to the magnetocaloric effect (MCE), analyzing the variation of magnetic entropy (*ΔS*_*M*_) as a function of temperature under various applied magnetic fields (μ_0_H), to further unravel the AFM-FiM magnetic order transition within the FCNO system. The MCE results (Figure 5) clearly show a temperature-dependent magnetic phase transition region (FiM-AFM). Similar behavior of MCE analysis has been previously reported for other systems, such as Ca_3_Co_2_O_6_^1^ and Sr_2‑x_La_*x*_CoNbO_6_,^35,55^ which exhibit geometrically frustrated spin chains and metamagnetic nature, due to the competition between FM and AFM interactions. In our case, $\Delta S_{M}^{min}$ = −0.13 J•kg^–1^•K^–1^ (at ∼ 32 K) can be attributed to the emergence of LRO, i.e., the AFM ordering state, while $\Delta S_{M}^{max}$ = 0.40 J•kg^–1^•K^–1^ (at ∼ 50 K) is associated with the FiM ordering state, which is the short-range ordering (SRO) state. As the temperature decreases below the region of maximum AFM ordering $\left(\Delta S_{M}^{min}\right)$ , the *ΔS*_*M*_ *(T)* curves become progressively intriguing. More precisely, at temperatures below ∼ 13.5 K, a further inversion in the *ΔS*_*M*_ *(T)* curves is observed, leading to the acquisition of positive values as the temperature decreases. This observation serves as a convincing indicator of a new magnetic transition occurring at T_t_ = 13.5 K. Consequently, there is a gradual evolution of short-range ordering within the incoherent chains as the temperature decreases. This progressive evolution results from the reestablishment of the FiM state, resulting from the FM interactions of the Fe sublattice (Fe^3+^–Fe^3+^) and the FiM interaction in the Fe/Cu sublattice (Cu^2+^–Fe^3+^), as confirmed in the Mössbauer spectrum obtained at 15 K. This explains the gradual increase in magnetization, as prominently illustrated in the M–T curve below approximately 13.5 K (see Figures 4b and 5b).

Based on the results of temperature-dependent magnetization, MCE, and Mössbauer spectroscopy data, a magnetic phase diagram as a function of temperature and its respective magnetic ordering states was constructed, as shown in Figure 8a. As previously discussed and now shown in the phase diagram, the FCNO remains in a FiM-1 ordered state up to ∼ 38.4 K, with 50 K being the temperature where the FiM order is maximized, corresponding to $\Delta S_{M}^{max}$ = 0.40 J•kg^–1^•K^–1^. Figure 8b illustrates a possible FiM configuration for the FCNO. Between 38.4 and 13.5 K the FCNO switches to an AFM-ordered state, with 32 K being the AFM ordering temperature maximized ( $\Delta S_{M}^{min}$ = −0.13 J•kg^–1^•K^–1^), as highlighted in the phase diagram and illustrated in Figure 8c. Below ∼ 13.5 K, the FCNO returns to the FiM-2 ordered state. However, with a different configuration, as depicted in Figure 8d. Above 50 K, thermal effects directly influence short-range intrachain interactions, which consequently lose magnetic ordering as the temperature increases. Thus, we must consider a PM contribution for *T* > 50 K, as already shown in the M-H curves [Figure 3(a)] and Mössbauer spectrum [Figure 4a]. The PM state arises from the loss of Fe^3+^-Fe^3+^ interaction due to temperature effects.

**Figure 8:**
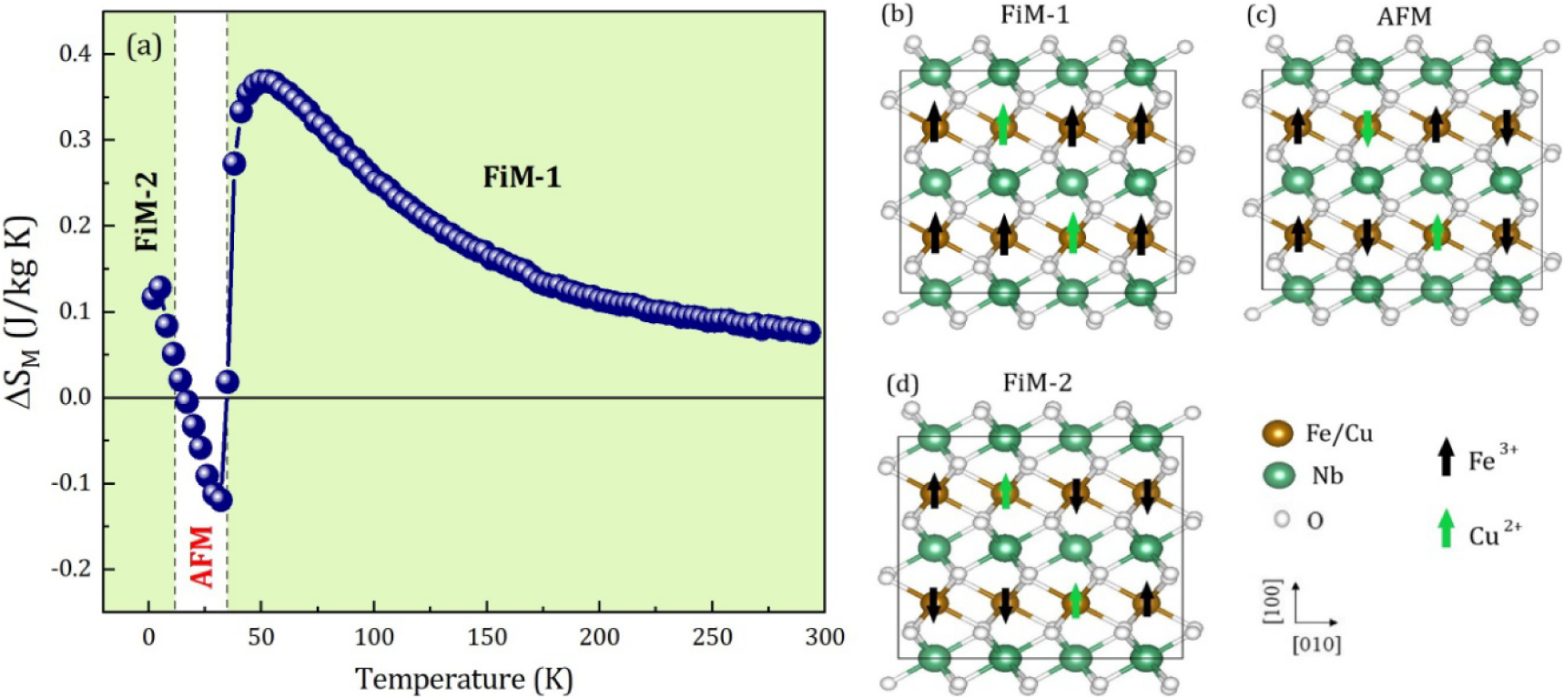
(a) Temperature-dependent magnetic phase diagram. Illustrative magnetic ordering configuration of the FCNO structure: (b) FiM-1 configuration, (c) AFM configuration, (d) FiM-2 configuration.

## 5 Conclusions

In summary, we have presented a comprehensive experimental discussion of the magnetism of the 1D-like FCNO chain system. We have verified the coexistence of multiple magnetic ordering states and spin reorientation processes originating from distinct sublattices. The FiM ordering state observed in FCNO originates from the Fe/Cu sublattice, which is characterized as localized magnetism. Moreover, we observed an antiferromagnetic T_N_ transition at 38.4 K and a T_t_ transition at 13.5 K by means of temperature-dependent magnetization measurements and magnetocaloric effect analysis, the latter associated with spin reorientation caused by the competition of FM and AFM exchange interactions, which was induced by Cu substitution on the Fe site. Interestingly, the transition observed below T_N_ is described as a localized transition originating from the Fe (Fe^3+^–Fe^3+^) sublattice, where the spins reorient again at *T* < T_t_. Finally, temperature-dependent Raman spectroscopic investigation reveals clear evidence of a magnetic transition followed by spin-phonon coupling in this compound, and no structural phase transition was measured between 10 and 300 K. Therefore, the present study provides a wealth of experimental results laying a solid foundation for future research efforts focusing on low-dimensional magnetic material systems.

## Supplementary Material



## Notes

Illustrative representation of the unit cell, Jahn–Teller effect and Fe–O–Fe bond angle for the compound Fe_0.75_Cu_0.25_NbO_4_; Peak fitting of the Cu LMM spectrum for the Cu-doped FeNbO_4_ sample; Peak decomposition of the Raman spectrum of the FCNO at room temperature and Table Raman-active modes and their identified assignments to structure (PDF)
