# Supplementary material for: Magnetic Phase Transition in the Quasi-One-Dimensional Spin Chain System Fe0.75Cu0.25NbO4
Source: ACS Omega. 2025 Dec 12;10(51):62645–56. doi: 10.1021/acsomega.5c06809 (PMC12756793; doi:10.1021/acsomega.5c06809)
Supplement: Supplementary file 1 [file ao5c06809_si_001.pdf]

Supplementary Material

Magnetic phase transition in the quasi-one-dimensional spin chain system $Fe_{0.75}Cu_{0.25}NbO_4$

Magnetic phase transition in the quasi-one-dimensional spin chain system $Fe_{0.75}Cu_{0.25}NbO_4$

Diego da Silva Evaristo*[1,2], Romualdo Santos Silva Jr[3], Raí Figueredo Jucá[1], Cledson dos Santos[1], Gilberto Dantas Saraiva[2], Javier Gainza[3], João Elias Rodrigues[4], Eva Céspedes[3], José Luiz Martínez[3], José Antonio Alonso[3], Igor Frota de Vasconcelos[5], Francisco Gilvane Sampaio Oliveira[5], Nilson dos Santos Ferreira[1], Antônio Joel Ramiro de Castro[6], Edson Caetano Passamani[7], and Marcelo Andrade Macêdo[1]

[1]Departamento de Física, Universidade Federal de Sergipe, 49100-000, São Cristóvão, SE, Brasil.

[2] Faculdade de Educação, Ciências e Letras do Sertão Central, Universidade Estadual do Ceara, 63902-098, Quixadá, CE, Brasil.

[3] Instituto de Ciencia de Materiales de Madrid (ICMM), CSIC, Cantoblanco, 28049 Madrid, Spain.

[4] European Synchrotron Radiation Facility (ESRF), 71 Avenue des Martyrs, 38000 Grenoble, France.

[5] Department of Engineering and Material Sciences, Technology Center, Federal University of Ceará, Campus do Pici, Bloco 729, 60440-900, Fortaleza, CE, Brazil.

[6] Universidade Federal do Ceará, Campus Quixadá, 63902-580, Quixadá, CE, Brasil.

[7] Departamento de Física, Universidade Federal do Espírito Santo, 29075-910, Vitoria, ES, Brasil.

* **Corresponding Author:** Diego da Silva Evaristo - Faculdade de Educação, Ciências e Letras do Sertão Central, Universidade Estadual do Ceará, 63902-098, Quixadá, CE, Brasil, daevaristo10@gmail.com

Data analysis-Rietveld refinement

The structural characterization was analyzed by the X-ray diffraction (Rigaku) using Cu-Kα radiation (λ = 1.54 Å) in the range of 10-70°, the step of 0.01° and counting time by step of 2s. The crystal structures lattice parameters were refined using the Rietveld method with the GSAS Software.[1]

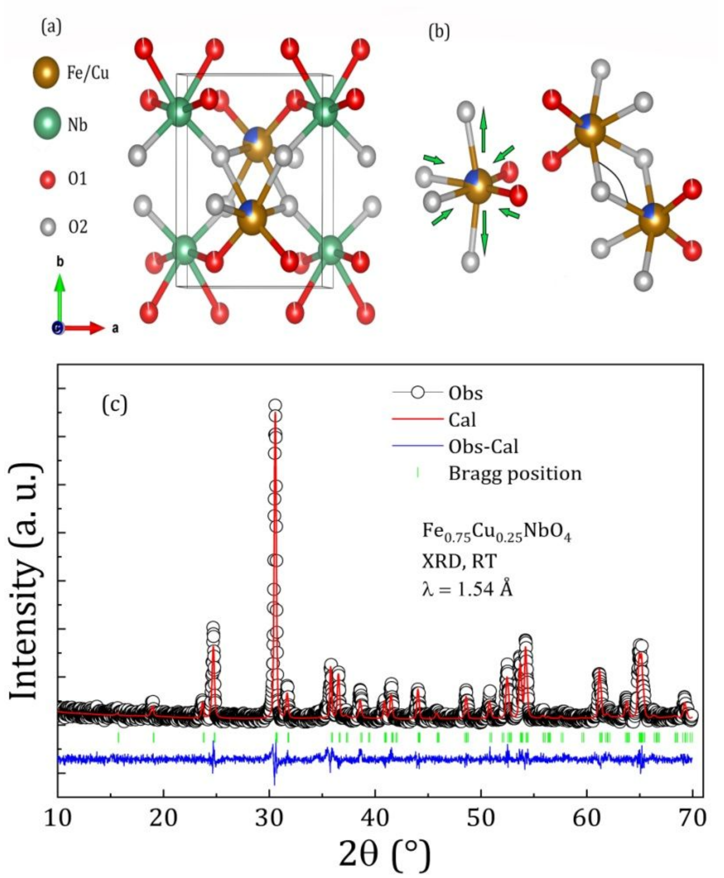

Figure S1: (a) Representation of the FCNO unit cell. (b) Jahn-Teller effect and Fe–O–Fe bond angle. (c) XRD Rietveld profile results at room temperature.

Table S1: Structural parameters at room temperature and quality factors.

Crystallographic data (space-group: P21/c)							
**Atom**	**Wyck. Posit.**	**x**	**y**	**z**	**Occ.**	**B**	**Mult.**
**Fe**	2*f*	0.5000(0)	0. 6734(3)	0.2500(0)	0.79(4)	0.524(1)	2
**Cu**	2*f*	0.5000(0)	0. 6734(3)	0.2500(0)	0.25(8)	0.524(1)	2
**Nb**	2*e*	0.0000(0)	0.1774(5)	0.2500(0)	1.00(0)	0.520(1)	2
**O1**	4*g*	0.2299(3)	0.1190(8)	0.5781(0)	0.97(0)	0.843(9)	4
**O2**	4*g*	0.2730(9)	0.3840(9)	0.0914(3)	1.00(0)	0.287(8)	4
**Lattice Parameters**							
a = 4.654(1) (Å)	b = 5.628(9) (Å)	c = 5.002(3) (Å)	V = 131.049(4) (Å^3)				
α = 90.00(°)	β = 90.06 (°)	γ = 90.00 (°)	ρ = 5.379(g/cm^3)				
Discrepancy factors: χ^2 = 1.41, R_{wp}= 22.01%, and R_p = 16.00%							

Energy Dispersive X-ray Spectroscopy- EDS

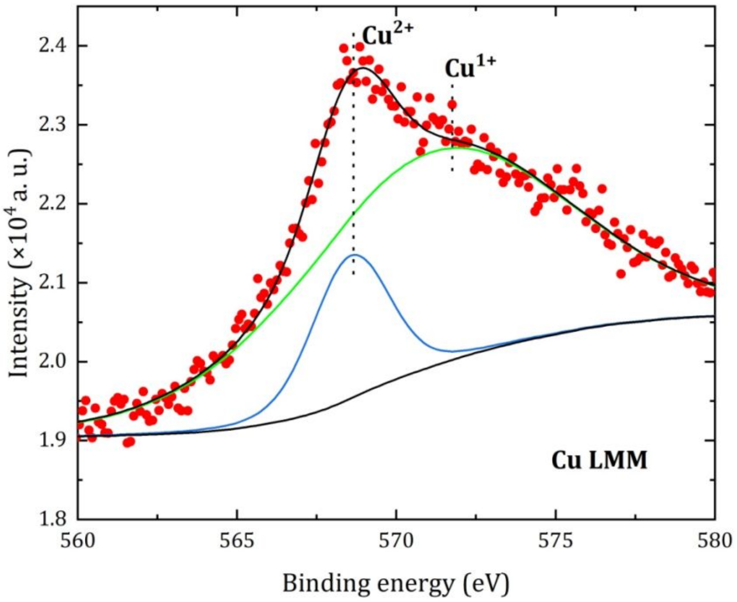

Figure S2. Peak fitting of the Cu LMM spectrum for the Cu-doped $FeNbO_4$ sample.

Raman spectroscopy and analysis as a function of temperature

Based on the analysis using the irreducible representations of the factor group (C_{2h}), the crystal symmetry inherent to FCNO implies that the *3N* degrees of freedom originating from the 12 atoms within the primitive cell are distributed across 36 vibrational modes, denoted as $\boldsymbol{\Gamma}_{\text{vibration}} = 8\mathbf{A}_g \oplus 10\mathbf{B}_g \oplus 8\mathbf{A}_u \oplus 10\mathbf{B}_u$. Within this set of 36 vibrational modes, 33 pertain to optical modes (k = 0), yielding $\boldsymbol{\Gamma}_{\text{optical}} = 8\mathbf{A}_g \oplus 10\mathbf{B}_g \oplus 7\mathbf{A}_u \oplus 8\mathbf{B}_u$, while the remaining 3 correspond to acoustic modes, expressed as $\boldsymbol{\Gamma}_{\text{acoustic}} = \mathbf{A}_u \oplus 2\mathbf{B}_u$. All these modes exhibit even vibrations (g), rendering them Raman active (18), whereas the odd vibrations (u) are declared IR active (15).[2,3,4,5] Upon decomposition of the room-temperature Raman spectrum using Lorentzian functions, we discerned the emergence of 16 distinctive vibrational bands at 138, 149, 173, 205, 223, 275, 301, 321, 363, 388, 413, 467, 496, 597, 742, and 827 cm^{-1}. These vibrational modes correspond harmoniously with the monoclinic FNO phase, as depicted in Figure S3.[2,3,4,5] The identification of each vibrational mode is briefly summarized in Table S1. The replacement of Fe^{3+} by Cu^{2+} ions within the ordered structure increases the cationic disorder, directly influencing the magnetic ordering.[6]

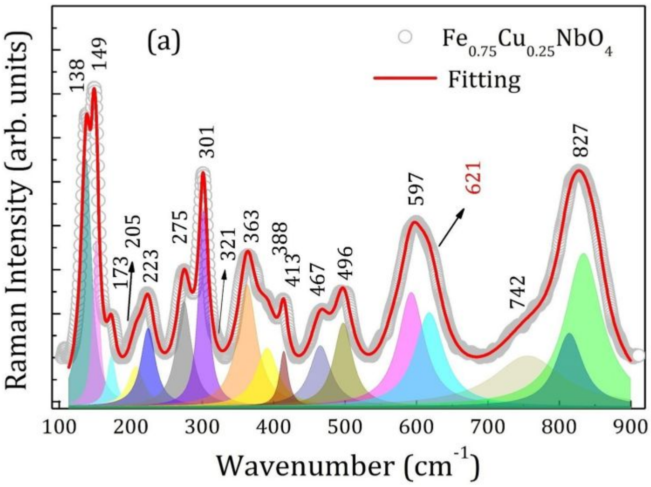

Figure S3: (a) Peak decomposition of the Raman spectrum of the FCNO at room temperature.

Table S2: Raman-active modes and their attributions identified through similar structure.[3]

Symmetry	ω_{obs} (cm^{-1}) 10 K	300 K	Assignments
B_g	137	138	Tx [NbO_6]
A_g	152	149	Ty[NbO_6 + FeO_6]
B_g	174	173	Txz [NbO_6 + FeO_6] in the diagonal of the xz plane
B_g	210	205	Txyz [NbO_6 + FeO_6]
B_g	227	223	Lib [NbO_6 + FeO_6]
A_g	277	275	Bend [NbO6 + FeO6] of strong motion of Fe atoms
A_g	303	301	Bend [NbO_6+ FeO_6] of moderate motion of Fe atoms along y axe
B_g	319	321	Bend [NbO_6 + FeO_6]
B_g	370	366	Lib [NbO_6 + FeO_6]
A_g	394	388	Bend [NbO_6 + FeO_6]
B_g	415	413	Bend [NbO_6 + FeO_6]
A_g	467	467	Bend [NbO_6 + FeO_6] strong motion of O in the Fe–O–Nb bonds
B_g	502	496	Bend [NbO6 + FeO6]
A_g	598	597	Symmetric stretching [NbO_6] + Bend [FeO_6]
Bg	623	621	Asymmetric stretching [NbO6] + Bend [FeO6]
B_g	747	742	Asymmetric stretching [NbO_6] + Bend [FeO_6]
A_g	827	827	Symmetric stretching [NbO_6] + Bend [FeO_6]

Bend: Bending, Lib: Libration, T: Translation.

References

(1) Toby, B. H. EXPGUI, a graphical user interface for GSAS, *Journal of Applied Crystallography*, 2001, *34,* 210-213.

(2) Liu, Y.; Wang, H.; Chen, G.; Zhou, D. Analysis of Raman spectra of $ZnWO_4$ single crystals, *Journal Applied Physics,* **1988**, *64*, 4651.

(3) Jucá, R. F.; Evaristo, D. S.; Oliveira, F. G.; Santos, L. P.; Saraiva, G. D.; Castro, A. J. R; A. J. R.; Ferreira, N. S.; Lobato, L. F.; Soares, J. M.; Brito, A. L. B.; Fausto, R.; Macêdo, M. A.; Costa, B. F. Co-doped $FeNbO_4$: Simple synthesis, DFT calculations and electrocatalytic performance for the hydrogen evolution reaction in alkaline medium, *Journal of Physics and Chemistry of Solids*, **2025**, *202*, 112649.

(4) Basiev, T. T.; Karasik, A. Y.; Sobol, A. A.; Chunaev, D. S.; Shukshin, V.E. Spontaneous and stimulated Raman scattering in ZnWO4 crystals *Quantum Electronics,* **2011**, *41*, 370-372.

(5) Ruiz-Fuertes, J.; Errandonea, D.; López-Moreno, S.; González, J.; Gomis, O.; Vilaplana, R.; Manjón, F. J.; Munoz, A.; Rodríguez-Hernández, P.; Friedrich, A.; Tupitsyna, I.; Nagornaya, L. High-pressure Raman spectroscopy and lattice-dynamics calculations on scintillating $MgWO_4$: Comparison with isomorphic compounds, *Phys. Rev. B,* **2011**, *83*, 214112.

(6) Evaristo, D. S.; Jucá, R. F.; Soares, J. M.; Silva, R. B.; Saraiva, G. D.; Matos, R. S.; Ferreira, N. S.; Salerno, M.; Macêdo, M. A. Antiferromagnet–Ferromagnet Transition in $Fe_{1-x}Cu_xNbO_4$, *Materials,* **2022**, *15*, 7424.
